# Investment incentive reduced by climate damages can be restored by optimal policy

**DOI:** 10.1038/s41467-021-23547-5

**Published:** 2021-05-31

**Authors:** Sven N. Willner, Nicole Glanemann, Anders Levermann

**Affiliations:** 1 Potsdam Institute for Climate Impact Research, Potsdam, Germany; 2 Columbia University, New York, NY USA; 3 Institute of Physics, Potsdam University, Potsdam, Germany

**Keywords:** Climate-change mitigation, Climate-change mitigation, Economics

## Abstract

Increasing greenhouse gas emissions are likely to impact not only natural systems but economies worldwide. If these impacts alter future economic development, the financial losses will be significantly higher than the mere direct damages. So far, potentially aggravating investment responses were considered negligible. Here we consistently incorporate an empirically derived temperature-growth relation into the simple integrated assessment model DICE. In this framework we show that, if in the next eight decades varying temperatures impact economic growth as has been observed in the past three decades, income is reduced by ~ 20% compared to an economy unaffected by climate change. Hereof ~ 40% are losses due to growth effects of which ~ 50% result from reduced incentive to invest. This additional income loss arises from a reduced incentive for future investment in anticipation of a reduced return and not from an explicit climate protection policy. Under economically optimal climate-change mitigation, however, optimal investment would only be reduced marginally as mitigation efforts keep returns high.

## Abstract (web summary)

Climate change is likely to damage economies worldwide. Here the authors show that this strongly reduces incentives to invest causing additional losses, whereas if investors include climate-change mitigation in their action portfolio they can avoid damages for themselves and the global economy.

## Introduction

With future emissions of greenhouse gases climate change is likely to impact not only natural systems^1–3^ but economies worldwide^4–7^. If these impacts alter future economic development, the financial losses will be significantly higher than the possible direct damages. Recent econometric analyses suggest that theses impacts may not just cause direct damage costs but decelerate economic growth and thus lead to persistent income losses in the future^8–11^. Such growth effects may significantly increase the total economic damage caused by climate change^12–17^.

**Fig. 1:**
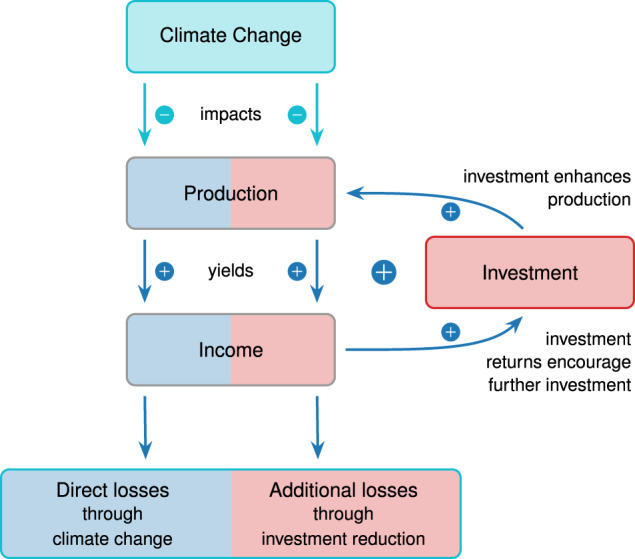
Illustration of the investment effect. Climate change reduces productivity, which translates into direct income losses (blue boxes). The prospect of reduced investment returns in the future renders investment less attractive. Accordingly, economically optimal investment is reduced and less production enhancing capital is accumulated. As a result, economic growth slows down and yields a future of persistently lowered income. This effect arising through reduced investment incentives is here referred to as the additional investment effect (depicted by red boxes).

A global analysis^9^ of the last three decades shows a maximum in the change of economic growth per capita at an annual average temperature of 13 °C. Increasing temperatures lead to a shift along this growth curve and overall yield a reduction in economic growth under future warming^9^, thus reducing production and income. A decline in productivity, i.e. the efficiency in transforming production input into goods and services, caused by temperature stress^9,18,19^ will evoke a response in investment behaviour. In general, it is to be expected that damages will reduce the incentive to invest and thereby lower the investment rate which will further reduce economic growth (Fig. 1). So far, this effect was suggested to be negligible^13^. However, other studies suggest a lasting effect of rising temperature on productivity as well as on asset valuations^20^.

Here we investigate the response of future economic investment as a central part of the growth effect under unmitigated climate change as well as under optimal climate policy. To this end we employ a standard economic growth model (DICE-2013R^21^), which is designed to compute the economically optimal investment strategy in a changing environment. These growth models frame the investment decision as an inter-temporal trade-off between present-day consumption and investment for production to enable more consumption in the future. It computes the investment path that is considered to be welfare-optimal by maximising the temporally aggregated societal value or utility of consumption. It is important to note that we do not claim that the results of our computation represent a projection of the actual future economic path. Instead we compute the optimal economic path under different assumptions. This path is optimal in the sense that it optimises the global utility of consumption. While we cannot claim that this is how the economy evolves, we can compare the resulting paths with and without climate damages and make a relative statement about the investment in both cases. This represents an estimate of the effect of climate change damages on future investment even in the absence of policy measures such as carbon taxes or a carbon trading scheme.

Here we show that, if in the next eight decades varying temperatures impact economic growth in the same way as has been observed in the past three decades, the economically optimal investment response almost doubles the income loss from climate-induced growth reduction. This additional income loss arises from a reduced incentive for future investment in anticipation of a reduced return not from an explicit climate protection policy. In computing the economic path that optimises this century’s global consumption under unmitigated climate change, we find a 22% income reduction compared to an economy unaffected by climate change. Hereof 40% are losses due to growth effects of which 48% result from a reduced incentive to invest under climate damages. On the other hand, economically optimal climate-change mitigation yields less than half the costs of unmitigated climate change. In this case, not only direct damages are reduced significantly, but also the effect of climate change on growth. As anticipated returns keep high, investment is only reduced marginally under climate abatement.

## Results

### Approach

**Fig. 2:**
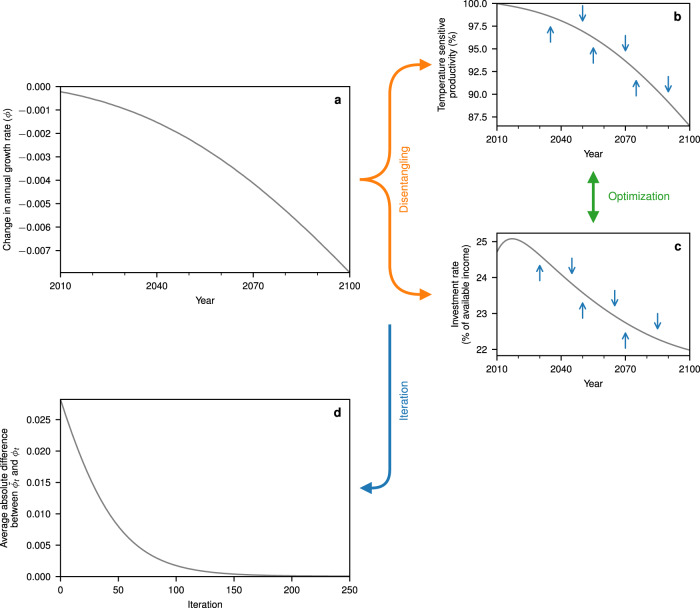
Schematic representation of the iterative procedure. The estimated change in the annual growth rate due to temperature increase^9^ (**a**) is disentangled into (**b**) the respective temperature-sensitive productivity function and into (**c**) its associated optimal investment response in the business-as-usual scenario, which is characterised by inaction of climate policy. **d** The iterated growth rate converges towards the estimated growth rate after ~200 iterations. Source data are provided as a Source Data file.

In light of recent empirical studies^8–11^ suggesting more considerable losses, we reconsider the role of the additional investment effect in exacerbating future income losses with and without climate-change mitigation. To this end, we modify the integrated assessment model DICE-2013R^21^ such that it accounts for the estimated global growth impacts^9^. DICE is based on a neoclassical growth model^22–24^, which computes economic growth effects caused by changes in investment. To preserve this feature, we develop an iterative process in DICE-2013R to find a productivity loss function that, taken together with the endogenously derived optimal investment response, reproduces the projected growth impacts in the absence of climate policy (Fig. 2; see Methods for more detailed information). This empirical productivity function yields direct damage costs of almost 10% of income for a warming of 3 °C compared to at most 5% in most previous studies^13,21,25,26^.

**Fig. 3:**
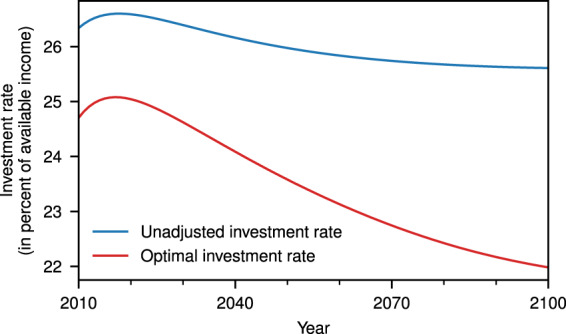
Comparison of the unadjusted and optimal investment rate. The optimal investment rate is significantly lower than the unadjusted version. Note that the unadjusted investment rate is the same for a scenario with climate change and one without. Source data are provided as a Source Data file.

With the iterative damage implementation in DICE-2013R, we compute the investment paths under different premises. ${I}_{\,\text{opt}}^{\text{nocc}\,}$ denotes the optimal investment in the absence of climate change; ${I}_{\,\text{unadj}}^{\text{cc}\,}$ denotes the investment in the presence of climate change but with unadjusted (i.e. same) investment rates compared to the case without climate change; and ${I}_{\,\text{opt}}^{\text{cc}\,}$ denotes the optimal investment under climate change in the sense as to maximise welfare. The optimal adjustment of investment to perceived climate damages already reduces the investment rate compared to a scenario with the absence of climate change significantly (Fig. 3).

### Investment response

**Fig. 4:**
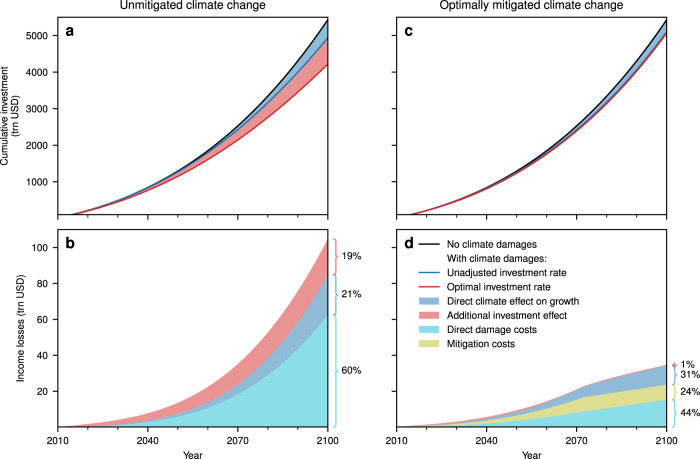
The growth effects in the absence of climate policy (a, b) and under economically optimal mitigation of emissions (c, d). **a** Unadjusted investment behaviour and particularly optimal investment lead to cumulative investment gaps through the climate effect on growth and the additional investment effect, respectively. **b** The income losses that occur for unadjusted investment behaviour (direct damage costs and the hereby induced growth effects) and for optimal investment (additional investment effect). **c, d** Economically optimal climate policy diminishes the climate effect on growth and renders the additional investment effect to be insignificant for (**c**) cumulative investment and for (**d**) income losses. Source data are provided as a Source Data file.

The optimal investment path under unmitigated climate change, ${I}_{\,\text{opt}}^{\text{cc}\,}$, yields a decrease in cumulative investment of 22% by 2100 compared to an economy without climate change, ${I}_{\,\text{opt}}^{\text{nocc}\,}$ (Fig. 4a). This leads to income losses over time (Fig. 4b) totalling to 104trn USD. The reasons for the income losses are (a) recurring direct damages caused by the warming that reduce the income available each year (63trn USD or 60%), (b) thereby slowed economic growth due to the reduced availability of investable income (22trn USD or 21%), and (c) the amplification of the decelerated economic growth through a reduced incentive to invest because of the anticipation of smaller future return of this investment (20trn USD or 19%); as illustrated in Fig. 1 by light blue, dark blue, and red shading, respectively. The influence of the investment reduction on the income loss is quantified by the comparison of cases with optimal investment rates (with and without climate change) with the case with unadjusted investment (${I}_{\,\text{unadj}}^{\text{cc}\,}$, red shaded area in each panel of Fig. 4).

**Fig. 5:**
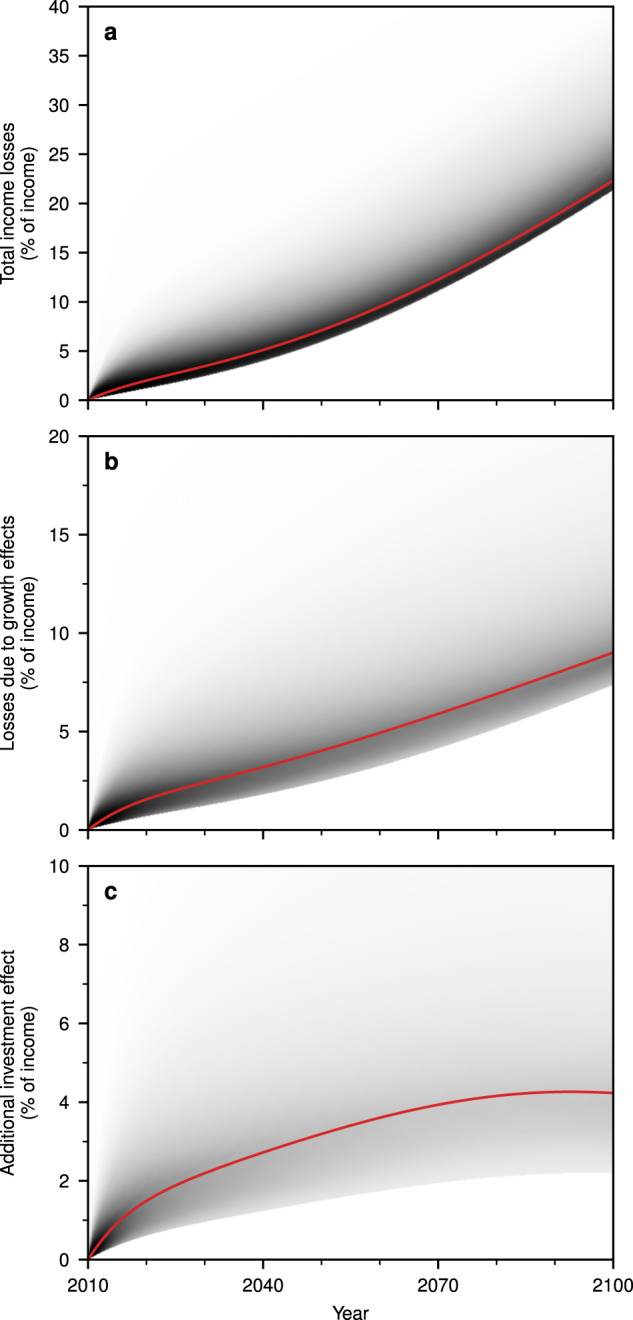
The influence of social preferences on the growth effects and income losses. Depicted are (relative to the income without climate change) the shares of (**a**) the total income losses; **b** the income losses caused through the growth effects; and (**c**) the income losses induced by the additional investment effect. Their magnitude depends on the social preferences. Solutions for alternative social preferences are illustrated by the grey area around the baseline solutions (red curves). The parameters are chosen uniformly within the unhatched area in Fig. 6. The shade of grey indicates the frequency with which the solution occurs. Source data are provided as a Source Data file.

The total income losses due to climate change without climate policy amount to 22% of the total income in 2100 (Fig. 5a). Hereof 40% are losses due to growth effects (9% of the total income, Fig. 5b). Of these growth effects 48% are due to the reduced investment (4% of the total income, Fig. 5c).

### The role of social preferences

As commonly applied in economic growth models^24^, the social preferences of consumption changes are represented by two parameters; the ‘initial rate of social time preference’ which expresses how strongly current consumption is favoured over future consumption and the ‘elasticity of marginal utility of consumption’ which captures the nonlinearity in the value of consumption for society (confer Methods for more details). We adhere to the original calibration of these parameters in DICE-2013R, which are chosen to resemble observed market interest rates to reflect plausible investment behaviour^26,27^ and provide a broad sensitivity analysis with respect to these normative parameters.

**Fig. 6:**
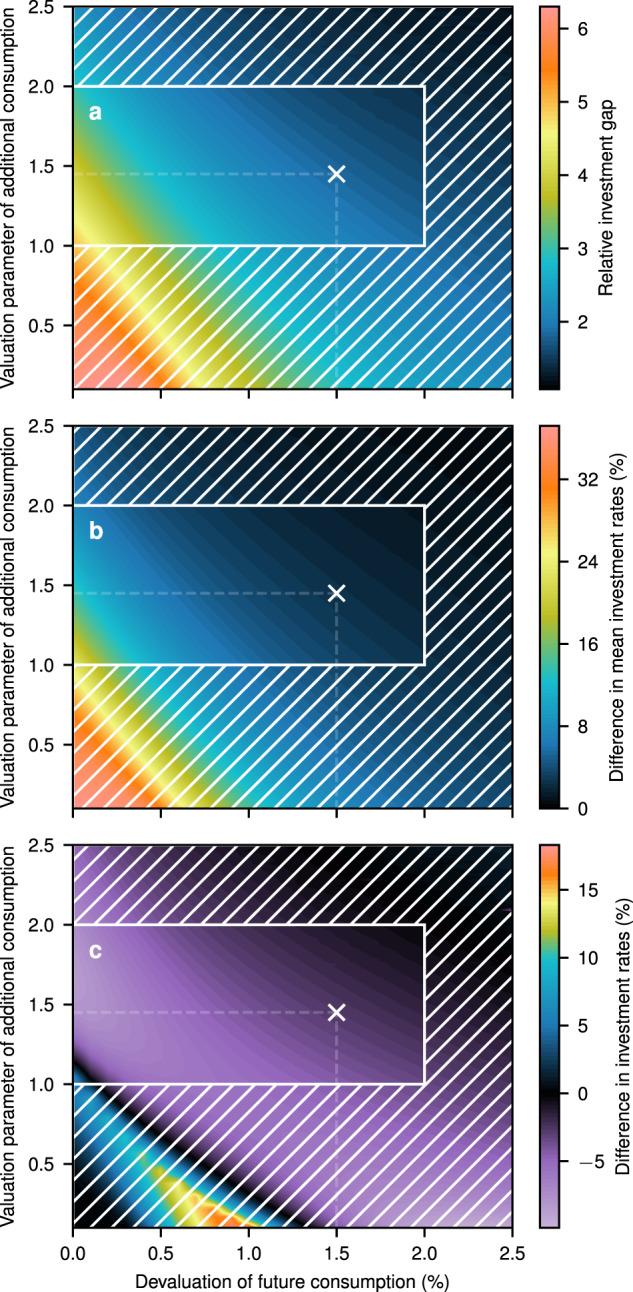
The effect of alternative social preferences. **a** The ratio of the investment gaps $\frac{({I}_{\,\text{opt}}^{\text{nocc}}-{I}_{\text{opt}}^{\text{cc}\,})}{({I}_{\,\text{opt}}^{\text{nocc}}-{I}_{\text{unadj}}^{\text{cc}\,})}$ in 2100. **b** Difference between the temporally averaged unadjusted and optimal investment rates of the years 2010–2100. **c** Difference in the optimal investment rate between 2100 and 2010. The unhatched area depicts the range as commonly used in the economic literature and the white marker indicates the baseline calibration. Source data are provided as a Source Data file.

Although the results are qualitatively the same for different values of these parameters, the magnitude of the investment effect varies significantly (Fig. 6, unhatched areas indicated values used in the economic literature^28^). For comparison we define the relative investment gap $\frac{\left({I}_{\,\text{opt}}^{\text{nocc}}-{I}_{\text{opt}}^{\text{cc}\,}\right)}{\left({I}_{\,\text{opt}}^{\text{nocc}}-{I}_{\text{unadj}}^{\text{cc}\,}\right)}$ (Fig. 6a). Positive values in 2100 suggest the existence of negative growth effects for a wide range of social preferences. For all considered preference combinations, we observe that the economically optimal decision is to reduce the investment rate (Fig. 6b). Furthermore, the optimal investment rate tends to decline over time for a large range of values (Fig. 6c). For very low values of the rate of social time preference (see Methods), the investment rate increases over time to counteract deficient consumption possibilities in the future.

### The role of mitigation

**Fig. 7:**
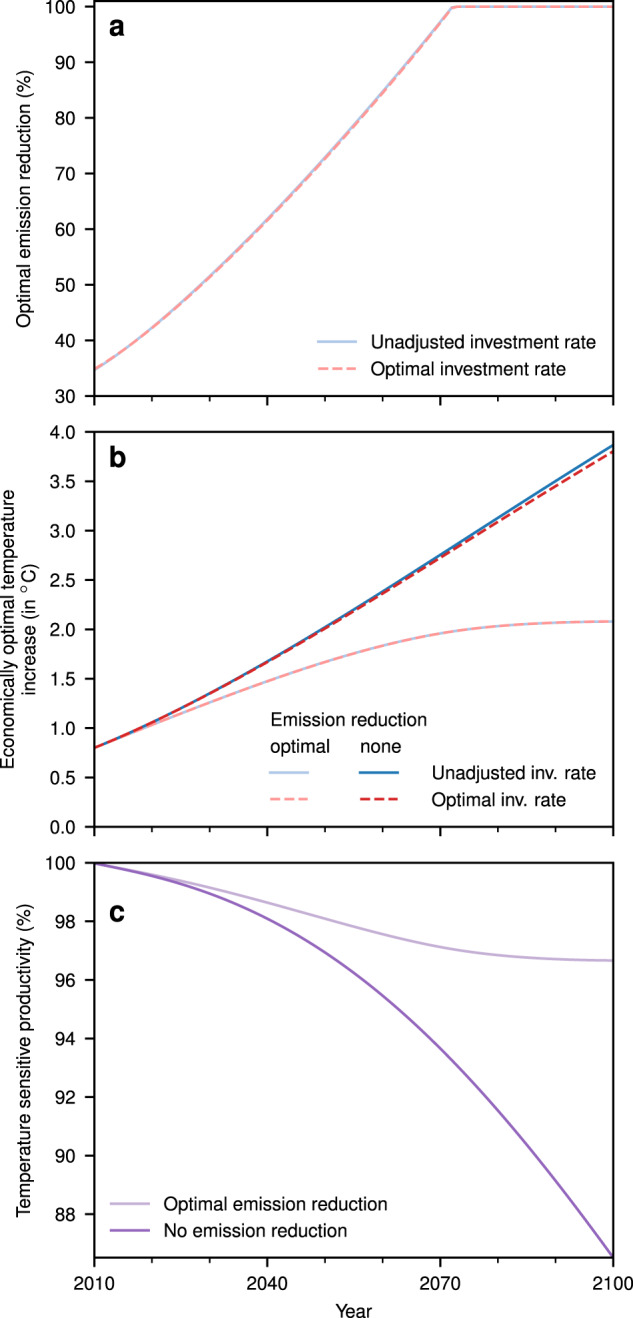
Optimal mitigation and its effects. **a** Optimal emission reduction rates are almost identical for the two assumptions of investment behaviour. **b** Economically optimal mitigation limits warming to almost 2 °C by 2100. **c** With unmitigated climate change, temperature-sensitive productivity decreases to ~86% by 2100, while economically optimal mitigation protects the economy from major productivity losses. Source data are provided as a Source Data file.

These computations compare different investment strategies without considering any policy to reduce carbon emissions. For comparison with the costs and benefits of climate-change mitigation, DICE-2013R allows to include the reduction of greenhouse gases as an additional means to maximise welfare. In that, mitigation reduces future emission intensity of production at the cost of present production. In computations that use this additional freedom of choice, no restriction on the global mean temperature is imposed, but the climate-induced damages yield an economically optimal warming around 2 °C compared to pre-industrial levels (Fig. 7b). We refer to these computations as climate-policy cases compared to the cases with unmitigated climate change.

To avoid future damages, in the optimal optimal climate-policy scenario emissions are cut back until they are completely phased out by 2070 leading to the strong limit in temperature increase (2 °C by 2100; Fig. 7a, b). As a consequence of this climate policy, temperature-sensitive productivity decreases only to ~97% of its value without climate change by 2100 (Fig. 7c), thus avoiding climate damages.

Whereas the reduction in investment in the unmitigated climate change scenario has only a small effect on temperature evolution—and thus only partially avoids damages—(Fig. 7b), reducing long-term emission intensity of production has a much larger effect. Accordingly, in the presence of an (optimal) climate-policy, the investment effect almost vanishes. Of the 35trn USD total income losses in 2100 only 1% (0.4trn USD) are due to investment reduction (Fig. 4d). Also, direct damages only total 15trn USD (44%) leading to growth effects of 11trn USD (31%). With the standard DICE mitigation cost function, mitigation costs sum up to 8trn USD or 24% of total income losses. Cumulative investment is thus reduced by only 6% through the direct climate effect on growth and 1% through the additional investment effect (Fig. 4c). As the optimal investment rate has only to be slightly adjusted in the climate-policy scenario, it also only has a negligible effect onto the optimal emission reduction (Fig. 7a). Thus, whereas the investment reduction under unmitigated climate change fails to properly avoid damages, proper mitigation does so quite well—keeping overall investment rates almost untouched.

The results of this paper can be summarised along the line of different economic response options to climate change. In the absence of climate change the investment growth would be strongest (black curve, Fig. 4a). Climate damages, however, reduce this investment by reducing the available capital (blue curve, Fig. 4a). The natural response of economic actors to the associated reduction in investment returns is to reduce the investment further (red curve, Fig. 4a). The additional investment reduction in response to the smaller anticipated returns is one and a half times the investment reduction due to the reduced capital from climate damages alone. This evolution corresponds to an adaptation-only perspective in which the economic actors simply respond to the climate damages without the perceived or real ability to mitigate climate change. It thereby depicts a guardrail for a possible future evolution. If, on the other hand, the ability to mitigate climate change is provided, on the economically optimal path the investment incentives stay almost as high as in the no-climate-change scenario and investment rates are only marginally reduced (red line, Fig. 4c).

## Discussion

Obviously the representation of the economic and the climate dynamics in the economic model applied here is very simple. It is however sufficient to provide an estimate of the optimal investment paths under a number of different assumptions. In particular it is assumed that the relationship between temperature and economic growth as found in the data for the years 1960–2010 remains a good approximation for the future^9^.

The incorporated investment decision rationale does not reflect any intention to reduce climate impacts for the purpose of the protection of society^29^. Instead it only aims at optimising utility of consumption. Thus, any reduced carbon emissions that might result from this optimisation are a reflection of the economic utility of such action. The decision rationale thus reflects a natural internalisation of the climatic externality without the use of globally coordinated policy instruments such as carbon pricing. That is because the climate-related growth reduction as applied here is derived from an observed relation between regional temperature and economic growth that does not result from a policy-driven internalisation of climatic damages, neither directly through compensation or indirectly through a carbon price. The only decision rationale that is reflected in our computations is that the investor has to decide how much to consume now and how much to invest for the future in order to maximise the utility of consumption. Under climate change economic productivity is reduced which means that more of the income is consumed and not invested because investment yields less return than in a world without climate change. As this is in a utility maximising context, keeping investments at the level in absence of climate change would actually be counterproductive and reduce utility—the returns of the additional investment are too low to balance the values that could not have been consumed earlier. Overall, the investment effect in our study is significantly higher than in previous computations because the observed climate impact on economic growth^9^ is larger than prior estimates.

Also, one must consider that DICE as a normative rather than descriptive model only yields paths optimal under its full constraints, in this case climate damages and mitigation. However, especially with difference in time scales between real-world investment and changes in climate impacts real-world investors might not follow this path. Though leading to smaller reduction in investment, this would mean larger damages and smaller returns on this investment.

We focus here on the direct damage costs, based on econometric analysis, and the associated investment response. Other effects that might become relevant in the future are thus not captured in this study. For instance, the investment effect could turn out to be less severe, if adaptation turns out to be more effective in protecting labour and capital productivity from warming than it was observed to be in the past. After all, the investment effect itself already is a form of adaptation, but alas not a productive one. The positive effect of adaptation can be, however, lessened or even reversed, if its financing requires withdrawing qlarge-scale amounts of resources from otherwise investable income. Lacking resources for research, product development, and education—whether caused by the growth effects discussed or by reallocation effects—can be another potential barrier to economic growth. Further growth effects, which are not captured by the growth projection used in this analysis, can stem from destruction of productive capital or from changes in the capital depreciation rate by climate-induced extreme weather events. Though these would be a potential target of additional investment its returns cannot be higher than when done in the absence of climate change. In the context of this study, replacement only occurs for missed production, not capital, and even that is limited by the anticipation of future damages, hence the investment effect.

Overall, our results stress that climate-change mitigation is in the strong interest of investors as the investment effect almost vanishes under optimal conditions. By contrast, continuing the business-as-usual path means either reducing investment in light of reduced marginal returns or risking additional missallocations with low returns that also reduce overall societal welfare.

We assume here that the observed climate impact on economic growth^9^ can be extrapolated into the future. This, however, neglects futher potential impacts such as high-order effects in the economic system^30–33^ or climate tipping points^34,35^. All these channels require in-depth research to gain a complete picture of economic climate impacts. In shedding light on the investment response, we aim to contribute here to the qualitative understanding of one piece of the puzzle.

## Methods

### General framework

In order to investigate the investment effect, we choose to transfer the recent climate-impact estimates by Burke et al.^9^ to the integrated assessment model DICE-2013R^21^.

Burke et al.^9^ estimate the relationship between temperature and changes in the development of economic growth based on observed data from 1960 to 2010. They present the results for individual countries (e.g. Extended Data Fig. 4 in Burke et al.^9^) and for the global sample (Extended Data Table 1 in Burke et al.^9^). They also compare data from 1960–1989 to 1990–2010 and find that this relationship has not changed significantly. Extrapolating this relationship into the future, they derive a future economic growth path under climate change.

In this growth path, direct productivity losses and the associated investment response are undistinguishable. The implementation of this growth path in DICE-2013R would thus turn it into an exogenous growth model, which has a possibly non-optimal investment path imposed upon externally. To maintain endogeneity of growth, we seek a productivity loss function in DICE-2013R that is consistent with the estimated growth impacts. For this, we take into account that the estimated relationship has not changed over the past decades and that it only applies where the fundamental dynamics resembles the one during the estimation period. These two aspects imply that, in order to disentangle productivity losses from growth effects, we have to impose assumptions about potential drivers of growth effects in the past. First, as resources spent on mitigation and adaptation have been rather small, growth effects that might be induced by reallocating investment resources for mitigation or adaptation purposes can be ignored; second, as the estimated relationship has not changed over time, notable adaptation was not induced and can thus be abstracted from; and third, the investment decision is sensitive to the emergence of future productivity losses, but the implications of the chosen investment path for future emissions and their accompanied climate-related impacts are not fed back into the decision making process. We believe that these climate considerations have not played a role for investment in the past. This is supported by the observation that the estimated relationship remained the same for several decades despite increased availability of information about the climate problem.

In order to analyse the additional investment effect, we include the resulting productivity loss function as the damage cost function in the original DICE-2013R version. We here follow Fankhauser and Tol^13^ and compare the income pathways for optimal investment and unadjusted investment behaviour that reflects ignorance of future productivity losses.

Note that, in contrast to our derivation of the direct productivity losses, which is based on a descriptive approach, we do not impose any additional assumptions on the investment decision. Whereas in the former case the investment decision is constraint by assumptions about past investment behaviour, in the latter case it accounts for all information and thus produces the economically optimal growth path.

### Climate impact projections

The temperature impact projections by Burke et al.^9^ describe future changes in observed levels of global income *Y* per capita *L* relative to a world with temperatures fixed at their 1980–2010 average. In particular, the evolution of income per capita is given as

1 $$\frac{{Y}_{t+1}}{{L}_{t+1}}=\frac{{Y}_{t}}{{L}_{t}}\left(1+{\eta }_{t}+{\phi }_{t}\right)$$

for all years *t*. Here *η*_*t*_ is the growth rate in the absence of climate change and *ϕ*_*t*_ the additional effect of warming on growth in that year. The growth rate *ϕ*_*t*_ is expressed in terms of a historical response function *h* as

2 $${\phi }_{t}=h\left({T}_{t}^{\,\text{ATM}\,}\right)-h\left({\overline{T}}^{\text{ATM}}\right),$$

with ${T}_{t}^{\,\text{ATM}\,}$ being the temperature in a given year *t* after 2010 and ${\overline{T}}^{\text{ATM}}$ being the average 1980–2010 temperature. The historical response function *h* is estimated as

3 $$h\left({T}_{t}^{\,\text{ATM}}\right)={\beta }_{1}{T}_{t}^{\text{ATM}\,}+{\beta }_{2}{\left({T}_{t}^{\text{ATM}}\right)}^{2},$$

with *β*_1_ = 0.0135 and *β*_2_ = − 0.0005. This calibration represents the main specification excluding data of countries with fewer than 20 years of growth data (Extended Data Table 1 in Burke et al.^9^).

It is important to remark that climate impacts on the economy are given here in terms of a growth rate. These growth effects need to be distinguished from damage functions that reduce the level of GDP. Typically, these level effect functions are relative productivity functions, which summarise the productivity reduction of labour and capital due to warming. As also explained by Burke et al.^9^, the standard Cobb–Douglas production function can be extended to account for temperature-sensitive labour productivity *A*^*L*^ and temperature-sensitive capital productivity *A*^*K*^ as

4 $${Y}_{t}={A}_{t}{\left({A}^{K}\left({T}_{t}^{\text{ATM}}\right){K}_{t}\right)}^{\gamma }{\left({A}^{L}\left({T}_{t}^{\text{ATM}}\right){L}_{t}\right)}^{1-\gamma }$$

5 $$=\underbrace{{A}^{K}{\left({T}_{t}^{{\rm{ATM}}}\right)}^{\gamma }{A}^{L}{\left({T}_{t}^{{\rm{ATM}}}\right)}^{1-\gamma}}_{{ = f\left({T}_{t}^{\text{ATM}}\right)}}{A}_{t}{K}_{t}^{\gamma }{L}_{t}^{1-\gamma }$$

6 $$=f\left({T}_{t}^{\,\text{ATM}}\right){Y}_{t}^{\text{gross}\,}\quad$$

with gross GDP at the beginning of the period ${Y}_{t}^{\,\text{gross}\,}$, temperature insensitive total factor productivity *A*_*t*_, productive capital *K*_*t*_, labour *L*_*t*_, output elasticity of capital *γ* and temperature-sensitive productivity $f\left({T}_{t}^{\,\text{ATM}\,}\right)$, $0\le f\left({T}_{t}^{\,\text{ATM}\,}\right)\le 1$. GDP net of level damage costs, *Y*_*t*_, can be considered to be the same as the observed income levels in Equation (1). One could similarly assume different temperature sensitivities as, for instance, temperature shocks can have a sizable impact on total factor and labor productivity^20^. Here, we only assume that, in its net effect, temperature acts onto gross GDP as ${Y}_{t}=f\left({T}_{t}^{\,\text{ATM}}\right){Y}_{t}^{\text{gross}\,}$.

### Transferring the growth estimates to DICE

We transfer the global growth impacts estimated by Burke et al.^9^ (Extended Data Table 1 in Burke et al.^9^) to the global model DICE-2013R^21^ with a simulation period corresponding to the projection period in Equation (2). For consistency with the estimated impacts, we also recalibrate this model to an annual time step version with 600 years by closely following the approach described by Cai et al.^36^. Furthermore, it is important to note that the warming effect in Equation (3) is expressed in terms of absolute annual temperature ${T}_{t}^{\,\text{ATM}\,}$, whereas in DICE-2013R temperature increase ${{{\Delta }}T}_{t}^{\,\text{ATM}\,}$ (in ^∘^ C from 1900) is considered. We thus convert temperature increase ${{\Delta }}{T}_{t}^{\,\text{AT}\,M}$ into absolute temperature according to

7 $${T}_{t}^{\,\text{ATM}\,}={{\Delta }}{T}_{t}^{\,\text{ATM}\,}-{{\Delta }}{T}_{2010}^{\,\text{ATM}}+{T}_{2010}^{\text{ATM}\,}$$

with ${{{\Delta }}T}_{2010}^{\,\text{ATM}\,}$ being the temperature increase in the initial simulation period, 2010. As the initial period might be unusually cold or warm due to variations in weather, we use the average temperature over 2005–2010 to calibrate the initial absolute temperature ${T}_{2010}^{\,\text{ATM}\,}$. The data for calibration is compiled from a NASA dataset^37,38^. The global average temperature increase in 2010, ${{\Delta }}{T}_{2010}^{\,\text{ATM}\,}$, stems from the original DICE-2013 version.

To implement the growth impacts, we disentangle the productivity loss function as described by Equation (4) from the investment response, which jointly cause the growth impact *ϕ*_*t*_. For this, we have developed an algorithm, in which we adjust the productivity loss function in DICE-2013R iteratively and solve for the optimal investment response. To be consistent with the assumption that growth effects induced by reallocating investment resources for mitigation or adaptation purposes can be ignored, we exclude the option to reduce emissions optimally. As stated above, we also assume that the investment decision process optimises the response to future productivity losses without account for its direct impact on emissions and consequent climate-induced damages. Essentially, this assumption is tantamount to postulating that the investment decision is made under ignorance of the temperature-productivity nexus. Accordingly, we seek a time-series *f*_*t*_, rather than a temperature dependent function, that fulfills

8 $${f}_{t+1}\frac{{Y}_{t+1}^{\,\text{gross}\,}}{{L}_{t+1}}=\frac{{Y}_{t}}{{L}_{t}}\left(1+{\eta }_{t}+{\phi }_{t}\right).$$

For *f* in the initial period we approximate ${f}_{1}\approx \left(1+{\phi }_{0}\right)\approx 0.99981$ with *ϕ*_0_ resulting from of Equation (2) with the temperature average of the preceding 5 years (2004–2009).

The iteration then proceeds as follows. We initialise the productivity with ${f}_{t}^{\left(1\right)}=1$ for all *t*, 1 ≤ *t* ≤ 600. For each iteration step *n* DICE-2013R finds an optimally chosen investment response to a given ${f}_{t}^{\left(n\right)}$. This yields the time series of income ${Y}_{t}^{gross,\left(n\right)}$ and ${Y}_{t}^{\left(n\right)}$. Further, investing according to the investment rate ${s}_{t}^{\,\text{nocc}\,}$ optimal in absence of climate change, ${I}_{t}^{\,\text{nocc}}={s}_{t}^{\text{nocc}\,}{Y}_{t}^{gross,\left(n\right)}$ yields the corresponding growth rate, ${\eta }_{t}^{\left(n\right)}$. Using the temperature time series ${{\Delta }}{T}_{t}^{ATM,(n)}$ we can, from Equation (2), derive the temperature-growth effect ${\phi }_{t}^{(n)}$ that follows the estimation of Equation (3). Equation (8) then provides a time series $\widetilde{{f}_{t}}$, which we use to update the productivity for the next iteration step,

9 $${f}_{t}^{(n+1)}={f}_{t}^{(n)}+\frac{\widetilde{{f}_{t}}-{f}_{t}^{(n)}}{2}.$$

The actual temperature-growth effect ${\overline{\phi }}_{t}^{(n)}$ in iteration step *n* as given by Equation (8) is sought to converge to that given by the estimation in Equation (2). Thus, the iteration algorithm is stopped once the time-average absolute deviation between ${\phi }_{t}^{(n)}$ and ${\overline{\phi }}_{t}^{(n)}$ has become sufficiently small (<6 ⋅ 10^−5^). At the same time, the optimal investment rate and the productivity function converge.

Eventually, the time series ${f}_{t}^{\left({n}_{\text{last}}\right)}$ and the temperature increase ${{\Delta }}{T}_{t}^{ATM,({n}_{\text{last}})}$ of the last iteration define the temperature-sensitive productivity function

10 $$f\left({{\Delta }}{T}_{t}^{\,\text{ATM}\,}\right):= {f}_{t}^{({n}_{\text{last}})},$$

in which we interpolate ${f}_{t}^{\left({n}_{\text{last}}\right)}$ linearly for the 600 sampling points of ${{\Delta }}{T}_{t}^{ATM,({n}_{\text{last}})}$. This function then replaces the damage cost function in the annual-period DICE-2013R model version.

### Background information on the social preferences

The preferences as displayed in Figs. 5 and 6 are represented by the initial rate of social time preference and the elasticity of the marginal utility of consumption. The initial rate of social time preference *ρ* is used to assign different weight to the utility *U* of per capita consumption ${c}_{t}=\frac{{C}_{t}}{{L}_{t}}$ at different time points $t\in \left[1,T\right]$ in the overall welfare function. In DICE, this social welfare function *W* is given by

11 $$W=\mathop{\sum }\limits_{t=1}^{T}{\left(\frac{1}{1+\rho }\right)}^{t-1}{L}_{t}U\left({c}_{t}\right).$$

In other words, *ρ* relates to impatience in consumption: a higher initial rate of social time preference gives more emphasis to present rather than to future utility. In such a case, society is inclined to consume more today and to invest less for future consumption possibilities.

The elasticity of the marginal utility of consumption *θ*, *θ* ≥ 0, determines the gain in utility due to additional consumption, irrespective of the timing of its appearance. It enters the utility function as

12 $$U({c}_{t})=\left\{\begin{array}{ll}\frac{{c}_{t}^{1-\theta }}{1-\theta }&{\rm{for}}\ \theta \ne 1\\ {\mathrm{ln}}\,{c}_{t}&{\rm{for}}\ \theta =1\end{array}\right.$$

The calibration of these parameters is controversially discussed in climate economics as they reflect either how decisions shall be formed on account of ethical concerns or how decisions are actually made. Ethical considerations are, for instance, reflected by an almost zero initial rate of social time preference, as it assigns future generations the same relevance as the current generation^39,40^. In contrast, the choice of a higher rate reflects that people usually prefer consuming today rather than postponing it. Likewise, the consumption elasticity parameter can be determined either based on empirical studies^28^ or by answering the normative question of how much importance additional consumption shall have for the society’s wellbeing^27^.

Together, these two parameters affect the trade-off in the allocation of available income between consumption and investment, and thus influence the additional investment effect.

## Data Availability

The source data underlying the figures are provided as a Source Data file. Source data are provided with this paper.

## Source data

Source Data

## Code availability

The code used in this study is available from the authors upon request.
